# Ions doped melanin nanoparticle as a multiple imaging agent

**DOI:** 10.1186/s12951-017-0304-3

**Published:** 2017-10-10

**Authors:** Shin-Woo Ha, Hee-Sang Cho, Young Il Yoon, Moon-Sun Jang, Kwan Soo Hong, Emmanuel Hui, Jung Hee Lee, Tae-Jong Yoon

**Affiliations:** 1 Medical Device R&D Center, Seoul National University Bundang Hospital, Seongnam, Gyeonggi-do 13605 South Korea; 2 Nanopharmacy Lab., College of Pharmacy and Research Institute of Pharmaceutical Science and Technology (RIPST), Ajou University, 206 Worldcup-ro, Yeongtong-gu, Suwon, 16499 South Korea; 3 Department of Radiology, Samsung Medical Center, Sungkyunkwan University School of Medicine, Seoul, 06351 South Korea; 4 Samsung Advanced Institute of Health Science and Technology, Sungkyunkwan University, Seoul, 06351 South Korea; 5 Bioimaging Research Team, Korea Basic Science Institute, Cheongju, 28119 South Korea; 6 Moogene Medi Co. Ltd., Gwankyo-ro 147, Gyeonggi-Bio-Center, Yeongtong-gu, Suwon, 16229 South Korea

**Keywords:** Melanin nanoparticle, MRI, CT, SPECT, Cancer imaging

## Abstract

**Background:**

Multimodal nanomaterials are useful for providing enhanced diagnostic information simultaneously for a variety of in vivo imaging methods. According to our research findings, these multimodal nanomaterials offer promising applications for cancer therapy.

**Results:**

Melanin nanoparticles can be used as a platform imaging material and they can be simply produced by complexation with various imaging active ions. They are capable of specifically targeting epidermal growth factor receptor (EGFR)-expressing cancer cells by being anchored with a specific antibody. Ion-doped melanin nanoparticles were found to have high bioavailability with long-term stability in solution, without any cytotoxicity in both in vitro and in vivo systems.

**Conclusion:**

By combining different imaging modalities with melanin particles, we can use the complexes to obtain faster diagnoses by computed tomography deep-body imaging and greater detailed pathological diagnostic information by magnetic resonance imaging. The ion-doped melanin nanoparticles also have applications for radio-diagnostic treatment and radio imaging-guided surgery, warranting further proof of concept experimental.
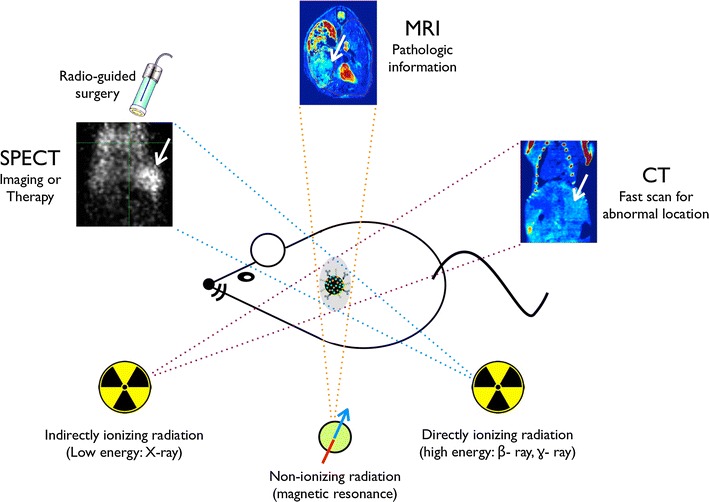

**Electronic supplementary material:**

The online version of this article (doi:10.1186/s12951-017-0304-3) contains supplementary material, which is available to authorized users.

## Background

Nanomaterials with a diameter between 1 and 100 nm show unique properties that are distinct from both molecules and bulk solids. Over the past decade, an explosive amount of novel nanoparticle-based imaging agents has been applied to bio-medical sciences [1, 2]. Magnetic, gold, radio-isotope embedded, and fluorescent nanoparticles have been developed as cancer-imaging contrast agents for non-invasive magnetic resonance imaging (MRI), X-ray computed tomography (CT), positron emission tomography (PET), single-photon emission CT (SPECT), and optical imaging [3–5]. However, most contrast agents only have a single imaging modality, which has intrinsic limitations such as insufficient sensitivity or spatial resolution, making it difficult to obtain accurate and reliable information at the disease site [6, 7]. To overcome these limitations, a multi-modal imaging material would be able to compensate for the limitations of materials with only a single imaging modality provide more detailed information on the interest site [8, 9]. For example, dual imaging data from MRI and PET can corroborate the pathologic state of muscle, vascular, and neural tissues, and accurately identify the location of abnormal tissues or cells in the body [10]. Although some multimodal imaging enabled inorganic particles have been developed, their application remains limited in the clinical setting because of complicated preparation steps and/or their acute toxicity potential [11, 12]. Naturally, organic nanomaterials, incorporated with imaging active ions are suggested as a superior imaging agent carrier. With regard to ions, Fe^3+^ can efficiently cause the longitudinal (*T*_1_) decrease of water protons for *T*_1_-weighted (*T*_1_-*w*) MR contrasting [13–16]. Bismuth (Bi), which has a relatively low toxicity, has been proposed as a good contrast agent candidate for indirectly ionizing X-ray radiation imaging tools such as CT due to its high density [17]. Directly ionizing gamma-radiation from radioisotopes such as ^99m^Tc can also provide real-time information during minimally invasive surgeries using a portable detector such as Geiger–Müller devices [18]. Furthermore, both beta and gamma-ray irradiation radionuclides like ^131^I can be simultaneously applied for radiotherapy by beta-ray irradiation and for conducting imaging diagnosis using the beta or gamma-ray by SPECT, gamma camera, or PET [19–21].

Here, we elucidate our findings on bio-inspired melanin nanoparticles (MNPs), which are optimized for cation loading, as imaging probes. The prepared multimodal imaging MNP platform doped simultaneously with Fe^3+^, Bi^3+^, and iodine was tested for improving the performance of triple imaging by MRI, CT, and SPECT. To increase biological efficiency, the MNP surface was chemically modified with a poly(ethylene glycol) (PEG) linker compound to enhance solution stability, and a cancer-specific antibody to actively target cells of interest. As proof of concept, the versatile ion-doped MNP (iMNP) was used against human hepatocellular carcinoma (HCC) target in vitro and in an in vivo system.

## Methods

### Synthesis and functionalization of iMNP

Dopamine hydrochloride (180 mg) was dissolved in 90 mL deionized water and then 1 N NaOH (780 μL) was added to the solution during vigorous stirring at 50 °C. The solution quickly turned dark brown. After 5 h, the black colored MNP solution was centrifuged at 18,000 rpm for 20 min and then washed several times with deionized water for elimination of excess chemicals. The MNP solution was then subjected to the ion doping process. To coordinate the Fe^3+^ ion, 4 mL Fe(NO_3_)_3_·6H_2_O (5.7 mM) solution was mixed with 10 mL of the MNP dispersion solution (5 mg/mL). After stirring for 10 min, the mixture were centrifuged (10 min at 20,000 rpm), and the unbound Fe^3+^ ions in the supernatant were quantitatively estimated in order to determine the overall loading capacity. Sequentially, Bi(NO_3_)_3_·6H_2_O (4 mL, 5.7 mM) and I–Cl solutions (50 μL, 8 mM) from the IODO-GEN^®^ (Additional file 1: Fig. S1) were mixed with Fe-doped MNP solution (5 mg/mL). Similarly, the treated particles were purified by centrifugation and washing with deionized water. The loading capacity and ion-releasing ability from the iMNPs were determined by measuring the ion concentration of the supernatant after centrifugation (20,000 rpm, 5 min) of iMNP solution by ICP-AES. For aqueous solution stability and selectivity, sulfhydryl (-SH) EGFR antibody (Abcam, Anti-EGFR antibody [2E9], ab8465) modified by Traut’s reagent was treated with the iMNP solution overnight at 4 °C. After centrifugation of the solution (5 min at 18,000 rpm) to remove unreacted antibodies, 2 mL PEG-SH (2 kDa, 0.5 mM) solution was added and reacted for 1 h at room temperature. After the reaction’s completion, the excess PEG-SH was removed by centrifugation (5 min at 18,000 rpm). The antibody from the iMNP-EGFR preparation was quantitatively analyzed for protein concentration by BCA protein assay. For the SPECT imaging study, borosilicate test tubes coated with 50 μg iodination reagent (IODO-GEN^®^, Thermo Scientific) were used for oxidation and incubated with 148 KBq of carrier-free Na^131^I (Korea Atomic Energy Research Institute) at room temperature until the color changed to yellow (~ 10 min). The solution was then added to a dispersion of Fe- and Bi-doped MNP (2.5 mg) in 10 mM HEPES with rapid agitation by vortexing. From this step forward, the samples were protected from light with aluminum foil. Unbound iodide-131 was removed by centrifugation (18,000 rpm, 15 min) and the precipitated product was dispersed in 1 mL PBS buffer. The fresh particle solution was administered to the mouse liver cancer model as soon as possible.

### In vitro phantom MRI/CT study

For the *T*_1_-*w* MR phantom study, various concentrations (0–9.6 mM, [Fe], [Bi], [Gd], or [I]) of iMNP-EGFR particles or Gadovist^®^ were prepared by introduction into a 0.5% agarose gel (1:1 volume ratio). To test in vitro phantom MR/CT imaging, a 3 μM concentration of the particles were incubated with various cells for 1 h inside a 5% CO_2_ incubator. The excess particles were removed gently by washing the cell culture with the culture media, and the treated cells were detached by trypsinization and subsequently centrifuged at 3000 rpm (5 min). All *T*_1_-*w* in vitro MR images were acquired on a 3.0-T clinical MR scanner (Philips medical system, Netherlands, archieva Release 3.2.1.0 version). Axial *T*_1_-*w* images were obtained with a TR at 400 ms, a TE at 10 ms, a 240 × 240 matrix, a flip angle at 90°, a slice thickness of 3 mm, number of averages with 4, bandwidth at 115 Hz/pixel, and an FOV of 120 × 120 mm. For CT phantoms, the used MR phantoms samples were transferred to the CT imaging scanner. The CT values (called Houndsfield units, Hus) with different concentrations of iMNPs were measured on an Inveon™ CT system (Siemens Healthcare, Germany). In vitro cellular CT imaging studies were performed using the above system as well as the equipment parameters of 80 kV and 400 μA.

### Cell toxicity assessment

A MTT assay kit (Invitrogen) was used to evaluate the cell viability after treatment with the MNP-based particles. The various cells were treated with various concentrations of the particles and culture times. Cells were maintained at 37 °C in a 5% CO_2_ incubator. The cells were washed, trypsinized, and re-suspended in the culture medium. The cells were then seeded at a concentration of 5000 cells/well in a 96-well tissue culture plate and allowed to grow overnight in a CO_2_ incubator. To determine the cell viability, the culture medium was replaced with the MTT solution. After 3 h of incubation inside a CO_2_ incubator, a specific MTT solution was added to dissolve the resulting formazan crystals. The cell viability was determined spectrophotometrically at 570 nm, with a background subtraction at 690 nm.

### Animal preparation

Male 6-week-old BALB/c nude mice were purchased from Orient Bio (Seoul, Korea). All animal studies were approved by the institutional Animal Care and Use Committee of Samsung Biomedical Research Institute (Seoul, Korea). Orthotropic liver tumor model was created using human hepatocellular carcinomas (HCC) liver tumor cell line (HepG2, Korean Cell Line Bank). After mice were anesthetized by inhaling 2% isoflurane in a mixture of O_2_/N_2_ gas (3:7 ratio) with a facemask, the liver was exposed. 1 × 10^6^ HepG2 cells suspended in 10 μL HBSS with Matrigel (1:1) were then slowly injected into the liver. After 4 weeks, tumor size was checked using MRI. The same anesthetic technique was used for obtaining MR images.

### In vivo MRI imaging

In this study, all mice were anesthetized using 2% isoflurane during the entire experiment (MRI, CT, and SPECT). MR images were acquired through a fast spin-echo *T*_1_-*w* MRI sequence by using the 7-T MRI system (Bruker Biospin, Fällanden, Switzerland), with the following parameters with respiratory gating: repetition time (TR)/echo time (TE) = 370/7.6 ms, echo train length = 2, 100 × 100 μm^2^ in-plane resolution with a slice thickness of 1 mm, and 14 slices. A quadrature volume coil (35 mm i.d.) was used for excitation and receiving the signal.

### In vivo Micro-CT/SPECT imaging

This study used the small animal integrated CT/SPECT imaging and analysis system (Inveon™ Micro-CT/SPECT multimodality system, Siemens Healthcare, Germany), which is designed as an in vivo system. The HCC liver implanted mice were intravenously injected with 200 μL of the iMNP-EGFR dispersion (148 KBq ^131^I, 0.3 μmol Fe, and 0.27 μmol Bi per 0.1 mg MNP/mL). All samples were scanned with 1.5 mm aluminum filter, using the following settings: 360° total rotation and 360 rotation steps, 1° step size, 70 kV and 400 µA source setting, and 700 ms exposure time per step. Pixels were binned by 4, resulting in an effective pixel size or resolution of approximately 74.93 µm. For each scan, the dataset was reconstructed with a down sample factor of 2 using the Inveon Acquisition Workplace software package (IAW, Siemens Medical Solutions, Knoxville, TN, USA), implementing the modified Feldkamp filtered back projection algorithm (Shepp-Logan filter). Afterwards, the reconstructed images were imported using the Inveon™ Research Workplace (IRW) into the accompanying two-dimensional (2D) and three-dimensional (3D) biomedical image analysis software package (IRW, CT Bone Visualization and Analysis, Siemens Medical Solutions, Knoxville, TN, USA) for visualization and analysis. For the SPECT imaging, SPECT scans were acquired using an Inveon™ micro-SPECT imaging system. SPECT images were obtained with high-resolution parallel hole collimators and the pulse-height analyzer window set over the 30 keV photopeak of iodine-131. In vivo CT imaging studies were performed using the same in vitro CT measurement parameters (80 kV and 400 μA).

### In vivo toxicological study

Male mice were housed in stainless steel cages containing sterile paddy husk as bedding in ventilated animal rooms. They were acclimated in the controlled environment (Temp.: 22 ± 1 °C, humidity: 60 ± 10% and light: 12 h light/dark cycle) with free access to water and a commercial laboratory complete food. The 100 μL PBS (control), MNP and iMNP-EGFR solutions were administrated daily via intraperitoneal injection for each of five mice with 0.5 mg concentration of particle after adaption. The treated mice were measured for body weight change over 15 days. After daily treatment for 2 weeks, the mice were harvested and various organs were analyzed for weight change and tissue morphology by hematoxylin and eosin (H&E) staining.

## Results and discussion

### Preparation of MNP and ions doping

**Fig. 1:**
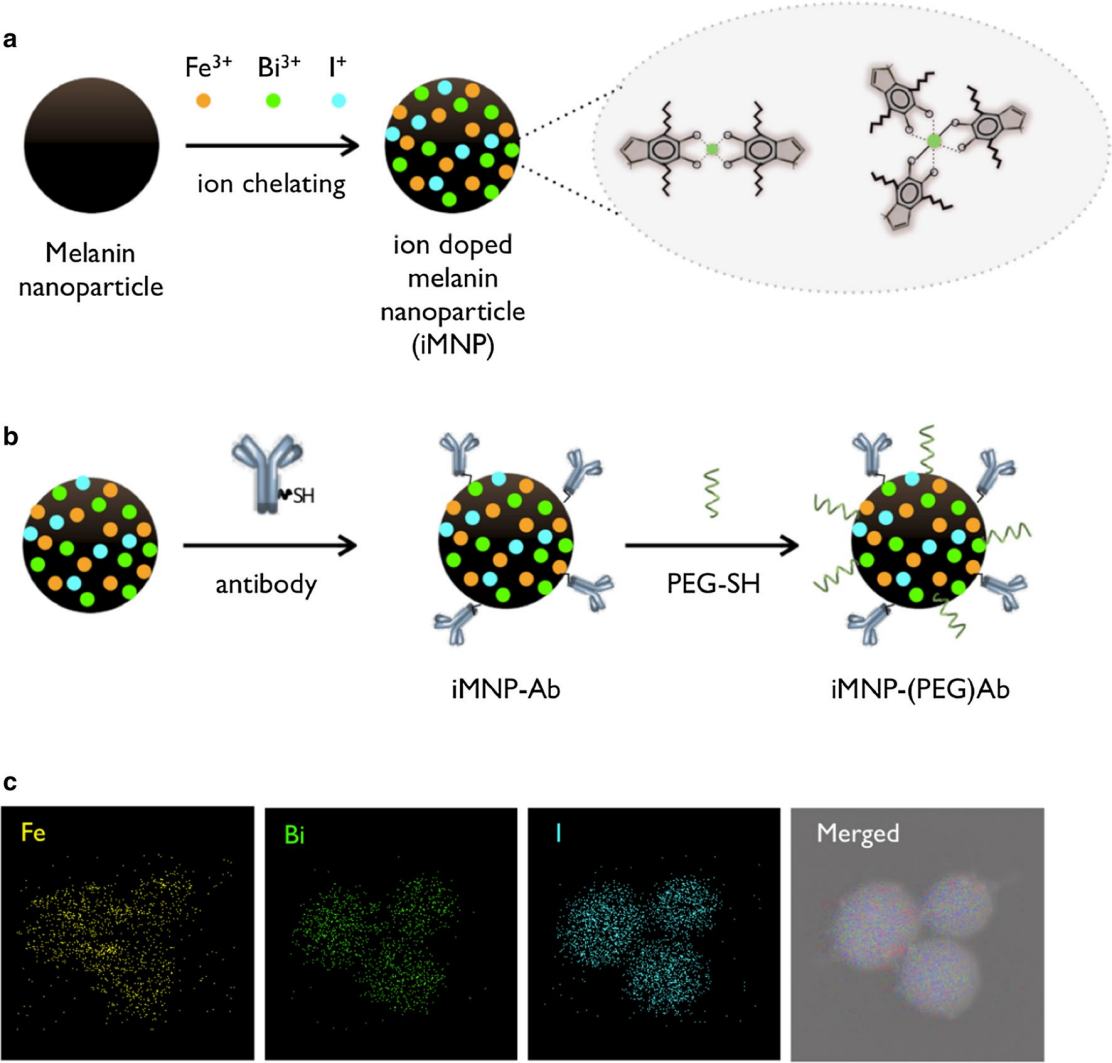
Schematic for the preparation of various ions chelating onto the melanin nanoparticle (iMNP, **a**), and surface modification with antibody and pegylation (**b**). Chelated Fe^3+^, Bi^3+^, and I^+^ ions were exhibited on the surface of iMNP by STEM (**c**)

**Fig. 2:**
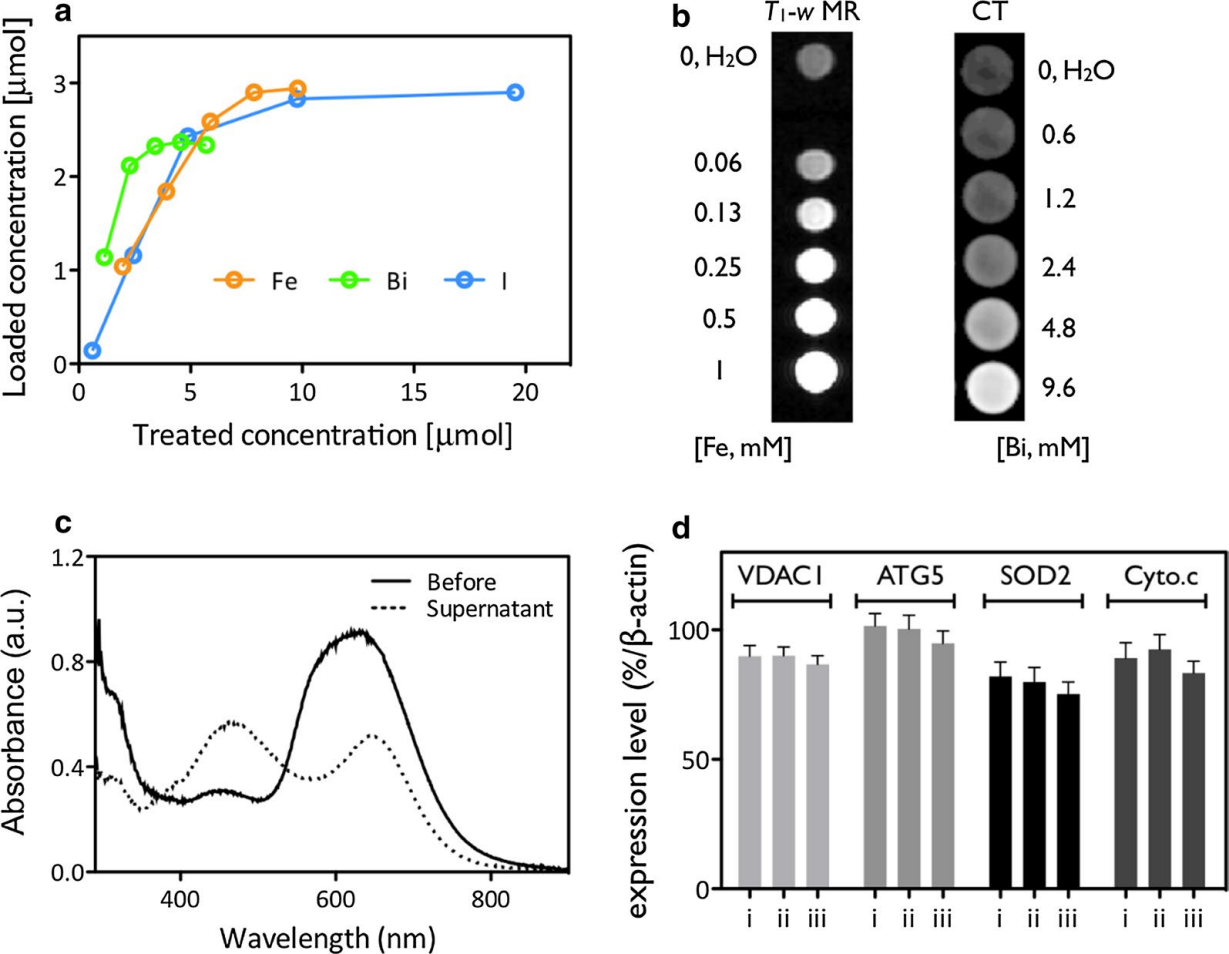
Chelating capacity of the various ions onto MNPs by ICP-AES analysis (**a**). *T*_1_-*w* MR (3.0-T, Philips) and CT (Inveon™, Siemens) contrast signal with different concentrations of ions (in iMNP) for sensitivity measurement (**b**). Protein quantification of antibodies conjugated onto the iMNP by BCA assay (**c**). Protein levels of various biomarkers concerned with cell organelle function or proliferation for measuring cytotoxicity after treatment by Western-blot analysis (**d**; i: only cell, ii: iMNP and iii: iMNP-EGFR; and see Additional file 1: Fig. S5)

The MNPs were synthesized successfully by neutralization and spontaneous air oxidation of dopamine hydrochloride as previously reported [22]. Briefly, 1 N NaOH solution was added into the dopamine hydrochloride solution under vigorous stirring to facilitate spontaneous oxidation and polymerization processes that formed the spherical MNPs. The particles were purified by washing with distilled water and centrifugation several times. From the transmission electron microscopy (TEM) analysis, the prepared MNPs showed an average diameter of 100 nm with a regular size distribution. The MNPs are comprised of 5,6-dihydroxyindole (catechol types) which were generated to form the coordination complexes with positive ions (Fig. 1a). Subsequently, sulfhydryl EGFR antibody and methoxy-poly(ethylene glycol) (PEG-SH) were introduced onto the particle surface by linkage via a Schiff’s base or Michael addition reaction (Fig. 1b) [23]. Using the MNP’s catechol groups, the Fe^3+^ and Bi^3+^ ions were simultaneously coordinated, and facilitated aromatic substitution reactions for I^+^ ion linkage onto the MNP surface. Morphology was characterized, and qualitative analysis was performed by scanning transmission electron microscopy (STEM). Generally, iodine radioisotopes (I*) such as ^125^I or ^131^I are purchased in their radio-iodide form, NaI*. However, the negative ion form of the radio-iodide chelated ineffectively with the catechol group of the prepared MNP due to charge repulsion. Therefore, we optimized the preparation of iodine MNPs using an iodination tube (Additional file 1: Fig. S1) that could produce the positive iodous ions (I^+^). Within 5 min, the NaI* (colorless solution) turned pale yellow (I*–Cl) and the positive iodous ions were easily substituted onto the MNP. From Fig. 1c, it can be seen that the various positive ions were present on the surface of the iMNP and were significantly different from the non-doped MNP as determined by energy-dispersive X-ray spectrometer (EDS) equipped STEM (Additional file 1: Fig. S2). To determine the loading capacity of the various positive ions, all ions were saturated at similar the concentration of 2.5–2.7 μmol in the 1 mL MNP solution (1 mg/mL) (Fig. 2a). Fourier transform infrared spectroscopy (FT-IR) was used to characterize the treated iMNPs by observing the change in the chemical functional group intensities during the ions complexation (Additional file 1: Fig. S3a). The *ν*_O–H_ group peak from the catechol decreased and reversely the *ν*_M–O_ showed increased vibrating peak intensity. The iMNPs also showed excellent dispersion ability in H_2_O, PBS buffer, culture media and human serum aqueous solutions for a month, validating their long-term stability in solution and suitability for biological applications (Additional file 1: Fig. S3b).

### In vitro phantom study for MRI and CT

Next, in a phantom study for imaging modalities, the iMNP was compared with commercial, clinically used *T*_1_-*w* MR (Gadovist^®^, Bayer Schering Pharma) and CT (Telebrix^®^, Guerbet Asia Pacific) imaging agents at the same concentration of ions. As a *T*_1_-*w* MRI agent, the iMNP solution displayed a 2-fold higher relaxivity than Gadovist^®^ by a 0.47 T magnetic relaxometer (mq-20, Bruker), and also, showed 4.5-fold higher Hounsfield (HU) intensity than the Telebrix^®^ solution at the same concentration of ions by an X-ray-based CT scan (Fig. 2b and Additional file 1: Fig. S4). From the comparative study of MR and CT phantoms, the superiority of the iMNP as an imaging agent was confirmed, as it can elicit the same signal strengths at 2-fold less than half of the equivalent dosage. According to the Solomon–Bloembergen–Morgan (SBM) theory predicting the efficiency of MRI contrast agents, longitudinal relaxivity (*r*_1_) is divided into two parts: inner sphere relaxation, which is influenced by directly coordinated water molecules, and outer sphere relaxation, which arises from interaction of the complex with water molecules in the second and outer sphere. Although the predominant effect of inner sphere relaxation is on *r*_1_ relaxivity, experimental studies on catechol–Fe^3+^ complexes of the iMNPs show that the interactions between second-sphere water molecules and oxygen atoms significantly contribute to their unusually high enhancing capabilities as well.

To generate specific targeting ability, epidermal growth factor receptor (EGFR) cancer cell specific antibodies were anchored onto the iMNP surfaces by antibody conjugation procedure, and the conjugated iMNP and EGFR antibody (iMNP-EGFR) preparation was quantified by a BCA protein assay (Fig. 2c). The doped ions did not leak from the iMNP under various pH aqueous conditions after 72 h of exposure, confirming the safety and reproducibility of the imaging agents (Additional file 1: Fig. S5a). Cellular toxicity of iMNP after uptake by EGFR-positive liver cancer cells (HepG2) was determined. The cell viability was over 90% under various concentrations and incubation times by 3-(4,5-dimethylthiazol-2-yl)-2,5-diphenyltetrazolium bromide (MTT) which is a colorimetric assay for assessing cell metabolic activity. Their changing expression levels monitored the various biomarkers for cellular functions after treatment with MNPs, iMNPs, and iMNP-EGFR particles by Western blotting. iMNPs caused a slight but in significant decrease in protein expression (Fig. 2d and Additional file 1: Fig. S5b and c).

**Fig. 3:**
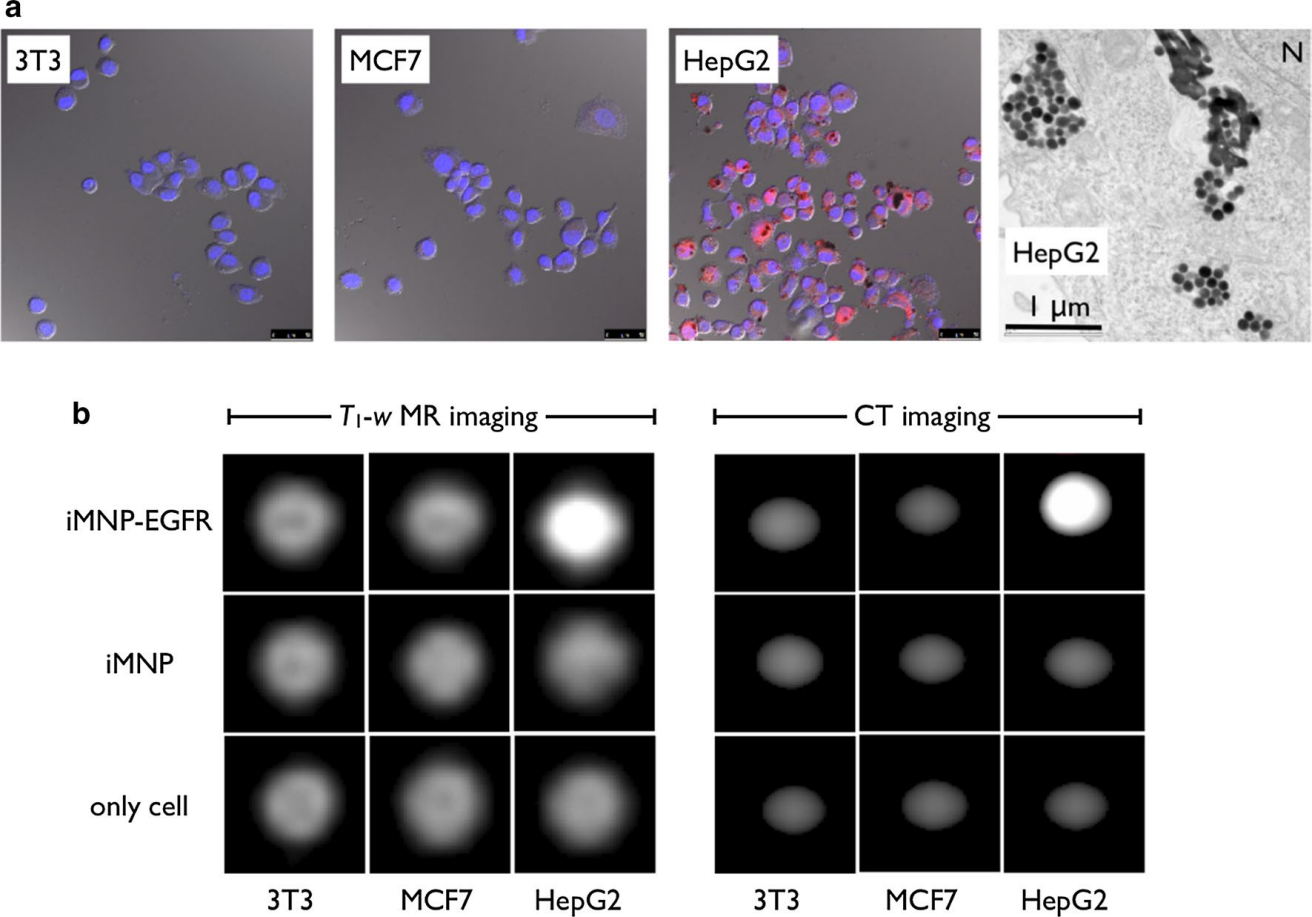
Specific targeting of the red-fluorescent iMNP after EGFR antibody conjugation (**a**, blue: DAPI nuclear staining and red: iMNP-EGFR, scale bar: 20 μm). Bio-TEM image of EGFR positive HepG2 liver cancer cell morphology after treatment with iMNP-EGFR (**a**, right, scale bar: 1 μm, N indicates the nucleus). Cellular phantom study data of the *T*_1_-*w* MR and CT imaging after treatment and accumulation of cells by centrifugation (see “Methods”) to confirm selectivity (**b**)

The iMNP-EGFR particles had high selectivity for the EGFR-positive liver cancer cells. Red fluorescent iMNP-EGFR, prepared after a conjugation reaction, was dominantly present in HepG2 cells, as opposed to EGFR-negative cell lines of NIH-3T3 and MCF7 (Fig. 3a and Additional file 1: Fig. S6). Moreover, a Bio-TEM analysis clearly determined that the iMNP-EGFR particles were distributed in the HepG2 cell cytosol presumably through energy dependent receptor-mediated endocytosis. The cells treated with various MNPs were used for *T*_1_-*w* MR (3.0-T, Philips) and CT imaging. In Fig. 3b, after treatment and making a cell pellet in a tube, EGFR-negative cells or non-antibody iMNP-treated cells showed similar low contrast intensity, but the iMNP-EGFR-treated HepG2 liver cancer cells exhibited a distinct high contrast signal.

### In vivo imaging

**Fig. 4:**
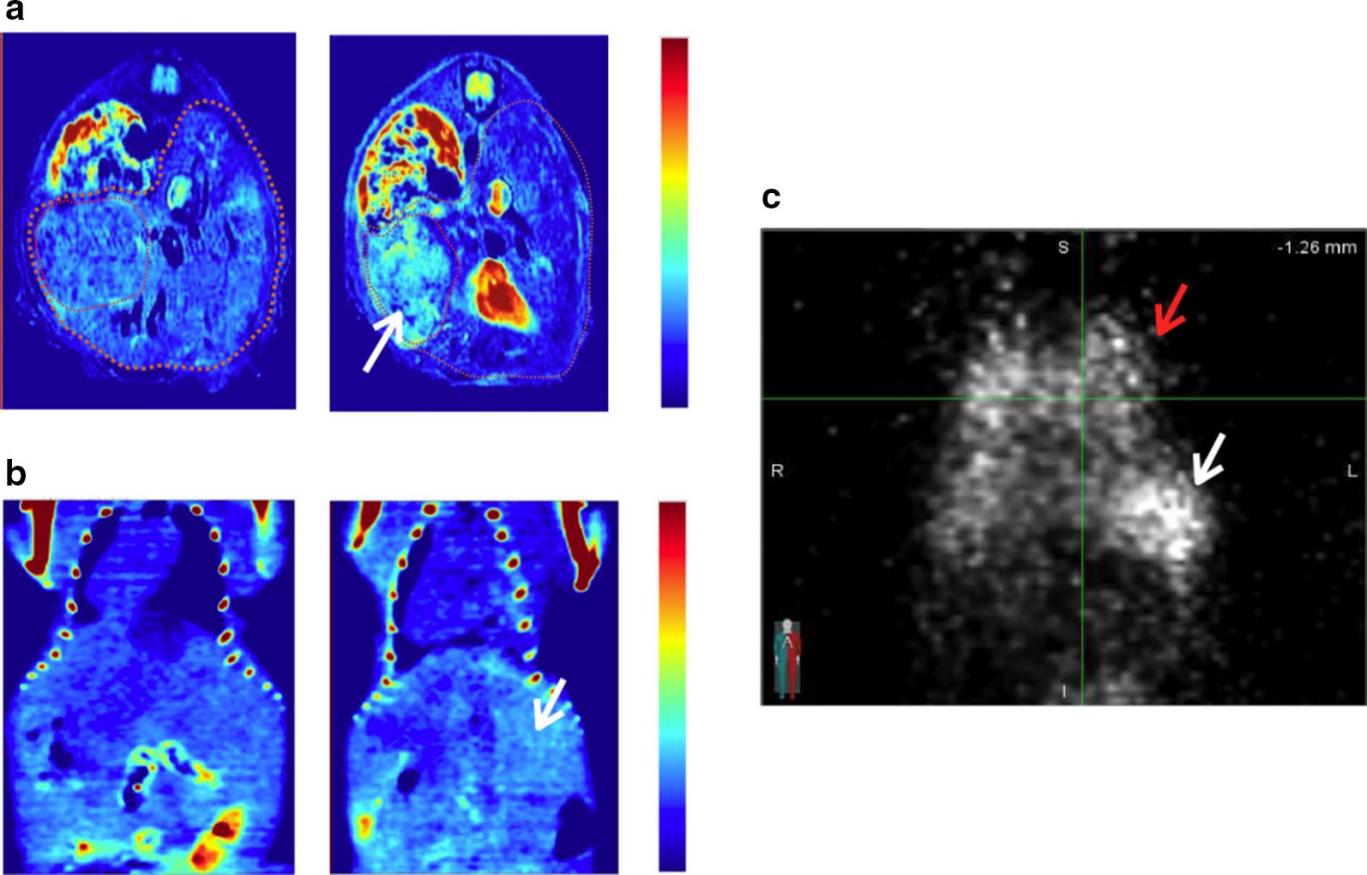
iMNP-EGFR mediated specific multi-modal imaging in in vivo cancer mouse model using various imaging tools after intravenous injection of the particles (**a** MRI, **b** micro-CT, and **c** micro-SPECT; see “Methods” section). The left images of **a** (axial view) and **b** (coronal view) indicate before administration of particle solution, and the right images of **a** and **b** are after 24 h of particle injection. In **c** (coronal projection view), the white arrow indicates the implanted HCC tumor in the liver and red indicates the normal lung

Next, we applied our iMNP-EGFR particles to an in vivo mouse tumor model. EGFR-positive HepG2 liver cancer cells were implanted into the liver, generating an orthotopic tumor model that is clinically more relevant than the subcutaneous implant model as a xenograft tumor model. During acquisition of MRI/CT/SPECT data all mice were anesthetizing by 2% isoflurane and the iMNP-EGFR particles were administrated through intravenous tail vein injection. In Fig. 4 and Additional file 1: Fig. S7, all MRI/CT/SPECT images exhibited high general contrast and comparatively the difference in specific targeting ability of iMNP-EGFR vs iMNP alone was expressed by concentrated particles in the liver tumor showing exceptional contrast. The preferential tumor accumulation of iMNP-EGFR particles resulted from the interaction of anchored antibody with tumor receptor as an active targeting and also the enhanced permeability and retention (EPR) effect during particle circulation. The individual images each have diagnostic meaning, and compensate for each method’s limitations, greatly improving diagnostic accuracy. Using multimodality offers the advantages of detailed cancer distribution information using SPECT imaging and clear location marking tumors using anatomical CT or MRI imaging. From these results, the rapid CT image suggested the possibility of a tumor with low sensitivity, but the additional MRI image identified the tumor in the liver with high resolution, and finally, the SPECT analysis provided detailed information about the clearance and distribution of particles. As a fundamental toxicological assessment, bare MNPs, iMNP-EGFR particles, and phosphate-buffered saline (PBS) were administered to normal mice by intravenous injection, and then monitored for body weight change and various organ functions (Additional file 1: Fig. S8). From the results, the MNP-treated mice did not show significant abnormality for body weight, but the color of several organs (especially liver, lung, and spleen) became black possibly due to the accumulation of the injected particles. However, after immunohistochemical (IHC) staining of the organ tissues, it was confirmed that the accumulated MNPs did not affect the tissue morphology (Additional file 1: Fig. S9). The iMNPs mainly accumulated in the liver, spleen, and lung, but the exposed organ tissues did not show any abnormality. Surprisingly, the iMNP-EGFR particles were slightly more concentrated in the lung than the other organs. Therefore, we will further investigate the mechanism of metabolism and interaction between melanin and lung tissue.

## Conclusion

The synthetic MNP platform, which is similar to that of living organisms like natural sepia melanin in cuttlefish, has the superior ability to simultaneous complex multiple types of positive ions. Moreover, MNPs can be simply fabricated and also have their surfaces modified with PEGylation and antibody conjugation. The doped metal ions were not released by biological environmental changes, and the iMNP-EGFR particles did not show significant cellular acute toxicity. From the phantom study, the iMNP-EGFR particle-imaging agent revealed higher contrast intensity than a clinically used imaging agent in *T*_1_-*w* MRI and CT. The EGFR antibody-conjugated iMNPs exhibited specific targeting to EGFR-overexpressed liver cancer cell in vitro, as observed by MRI and CT imaging. The iMNP-EGFR particles were applied for the simultaneous multi-imaging of xenograft liver cancer and the successful targeting of EGFR-expressed orthotopic tumor in mouse models. Most importantly, administration of the iMNP imaging contrast agent could be clinically applied for the rapid diagnosis of cancer by CT scanning. Detailed information can also be gathered by MRI for discovering and addressing morphologically and functionally pathophysiologic questions. Furthermore, as a proof-of-concept experiment, radio imaging using the iMNP system exhibited the possibility for its radio-therapeutic application by beta-ray irradiation or radio-guided surgical treatment using detector tubes. Our findings suggest that the iMNP system will be a suitable candidate probe for various diagnostic imaging modalities and radio-guided treatment tools. In a future study, we will perform a detailed in vivo toxicological assessment of the iMNPs’ pharmacokinetics, lethal dose calculation, and the half maximal effective concentration (EC50) in preclinical practice.

## Additional file

**Additional file 1.** Iodination of MNP using an IODO-GEN tubes. STEM analysis data for ionized MNPs. Characterization data for iMNPs. Phantom study data for iMNPs in solution. Cellular toxicity assessment after iMNP treatment. Characterization data for the expression levels of EGFR. In vivo toxicological data after administration of iMNPs.

## Abbreviations

EGFR: epidermal growth factor receptor
MRI: magnetic resonance imaging
CT: X-ray computed tomography
PET: positron emission tomography
SPECT: single-photon emission CT
MNP: melanin nanoparticle
iMNP: ion-doped MNP
PEG: poly(ethylene glycol)
HCC: hepatocellular carcinoma
STEM: scanning transmission electron microscopy
EDS: energy-disperse X-ray spectrometer
FT-IR: Fourier transform infrared spectroscopy
