# Supplementary material for: Ions doped melanin nanoparticle as a multiple imaging agent
Source: J Nanobiotechnology. 2017 Oct 10;15:73. doi: 10.1186/s12951-017-0304-3 (PMC5635546; doi:10.1186/s12951-017-0304-3)

**Supplementary Materials**

Ions doped melanin nanoparticle as a multiple imaging agent

Shin-Woo Ha^1†^, Hee-Sang Cho^2†^, Young Il Yoon^1^, Moon-Sun Jang^3^, Kwan Soo Hong^4^, Emmanuel Hui^5^, Jung Hee Lee^3*^ and Tae-Jong Yoon^2,5*^

^1^ Medical Device R&D Center, Seoul National University Bundang Hospital, Seongnam, Gyeonggi-do, 13605, South Korea

^2^ Nanopharmacy Lab., College of Pharmacy and Research Institute of Pharmaceutical Science and Technology (RIPST), Ajou University, 206 Worldcup-ro, Yeongtong-gu, 16499 South Korea Suwon, E-mail: tjyoon@ajou.ac.kr

^3^ Department of Radiology, Samsung Medical Center, Sungkyunkwan University School of Medicine, Seoul 06351, South Korea; Samsung Advanced Institute of Health Science and Technology, Sungkyunkwan University, Seoul 06351, South Korea, E-mail: hijunghee@skku.edu

^4^ Bioimaging Research Team, Korea Basic Science Institute, Cheongju 28119, South Korea.

^5^ Moogene Medi Ltd., Gwankyo-ro 147, Gyeonggi-Bio-Center, Yeongtong-gu, Suwon 16229, South Korea.

**Figure S1.** Iodination of MNP using an IODO-GEN^®^ tube (Pierce). To chelate ions onto the MNP, the ion should have a positive charge because of the interaction with o-dihydroxyl group of the MNP catechol. We could generate the active iodous ion (I^+^) form through an optimized process. Colorless NaI aqueous solution (50 μL, 8 mM) was added into the tubes (PBS 90 μL) and stirred vigorously. After 3 min, the color turned pale yellow indicating the formation of I-Cl in solution. Subsequently, MNPs containing Fe and Bi ions on their surfaces (5 mg/mL, 50 μL) were dispersed directly into the tube solution and incubated for 30 min. After that time, the iMNPs were centrifuged and then re-dispersed in PBS before the next process. The iodination of iMNPs was monitored by I-Cl absorption UV-Vis spectra (below).


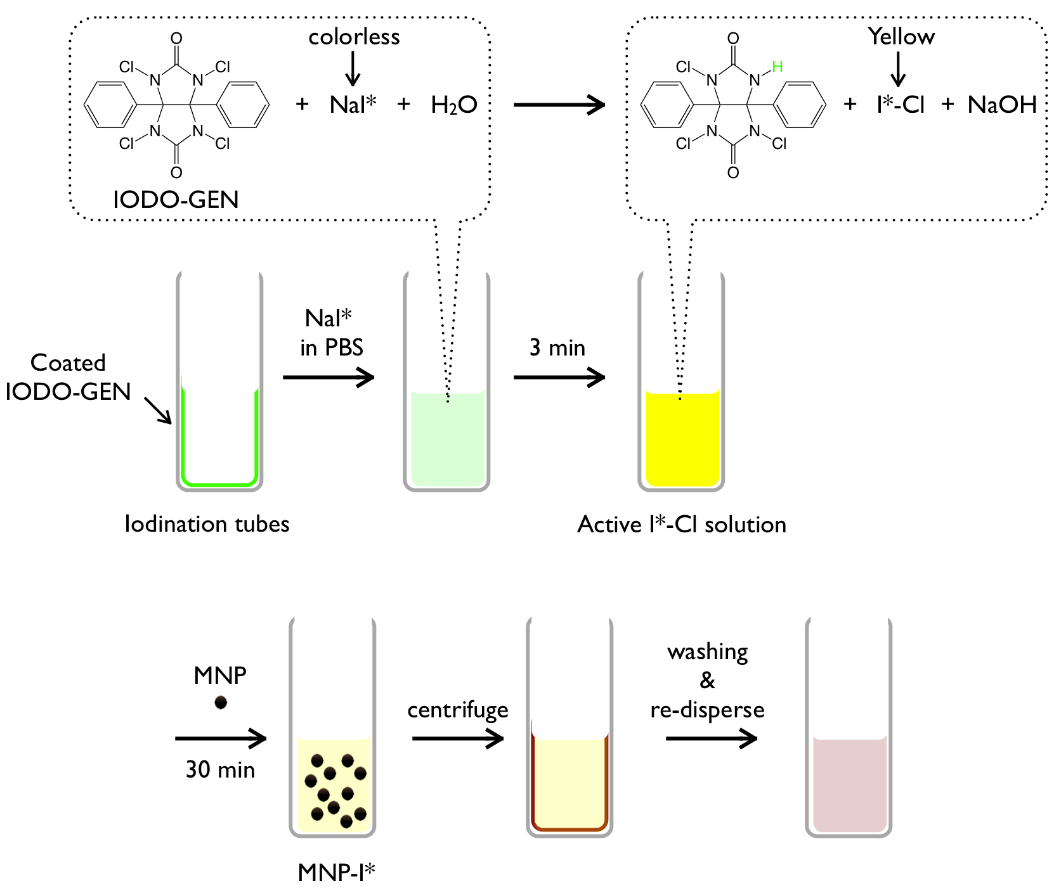


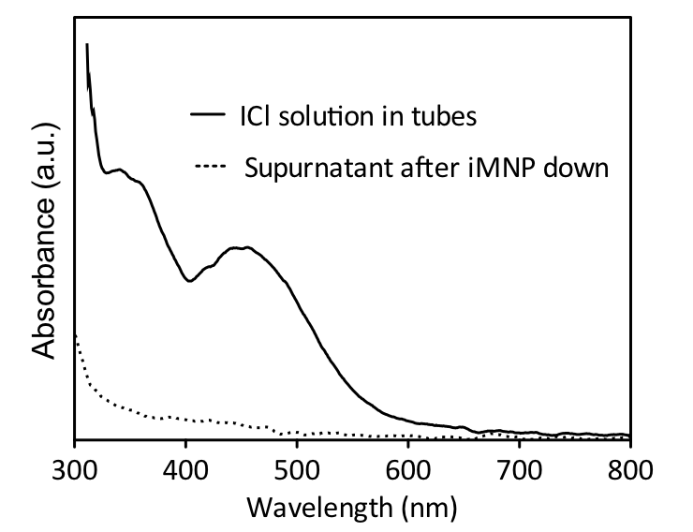


**Figure S2.** The surface of pure MNP without any chelated ions was characterized by EDS equipped STEM (A) and showed no ion-specific signal intensities. After chelating various ions (Fe, Bi, and I), the iMNP exhibited exact ions peaks in the EDS analysis (B).


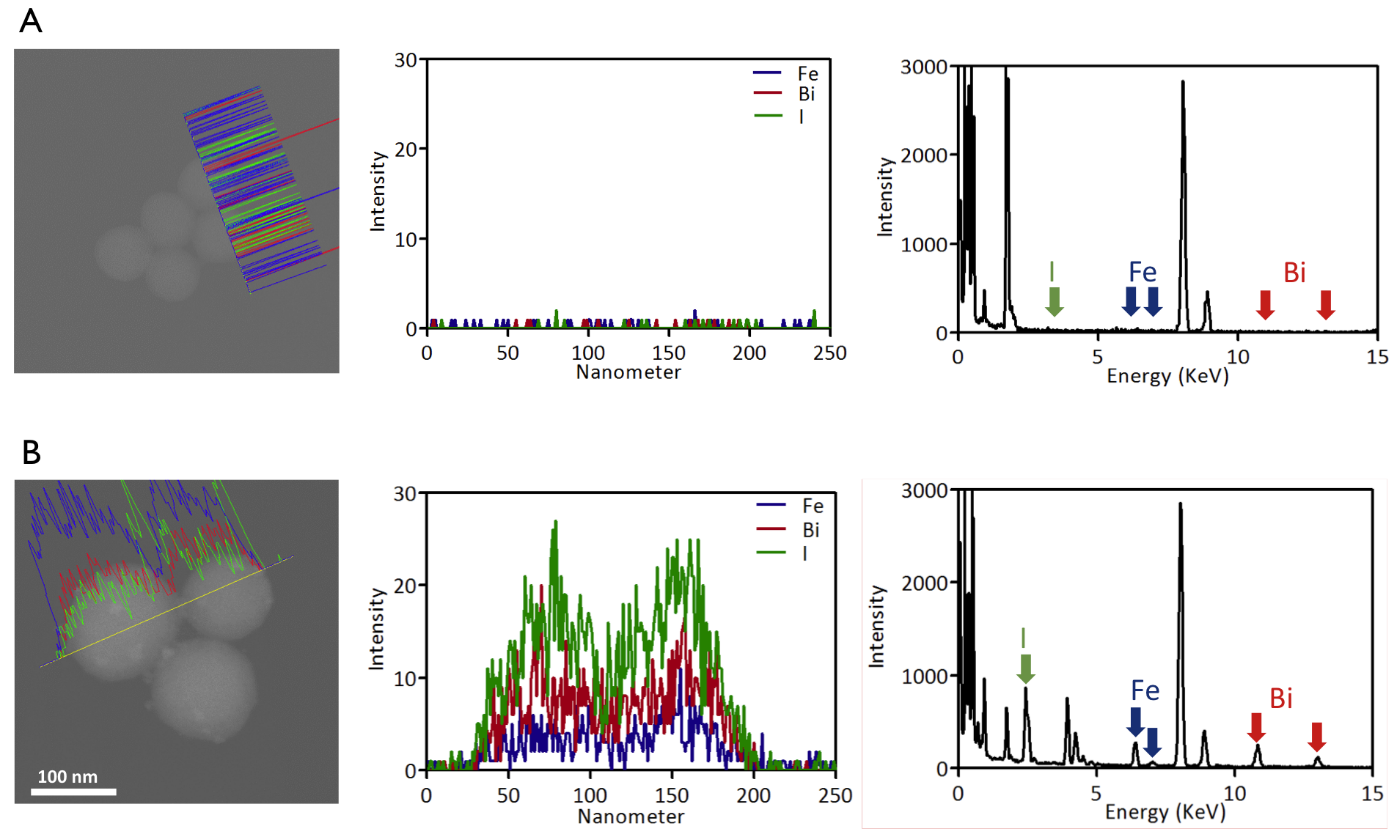


**Figure S3.** Changing of MNP surface chemical functional groups after doping was measured by FT-IR (A). The O-H peak decreased, and the metal oxide peak increased in relative intensity after treatment with the ions. The iMNP showed long-term stability (~ 30 days) in solutions without self-aggregation by DLS study which the dispersion solutions were purchased from Sigma-Aldrich (B).


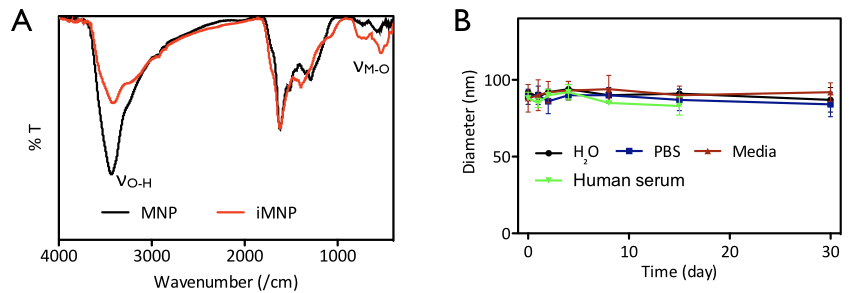


**Figure S4.** Sensitivity measurement of iMNP in *T*_1_-*w* MRI and CT as a phantom study. The longitudinal relaxivity (*r*_1_) value of the prepared iMNP-EGFR particle and a clinically used T1 MRI contrast agent (Gadovist^®^, GDV) using a 0.47 T magnetic relaxometer (mq20, Bruker) (A). The relaxation time (*T*_1_) was detected at various concentrations of metal ion, which was calculated accurately using ICP-AES analysis after measurement. The iMNP chelated with Fe ions solution exhibited a ~2-fold higher *r*_1_ value (6.2 s^-1^·mM[Fe]^-1^) than Gadovist^®^ (3.2 s^-1^·mM[Gd]). In terms of *r*_2_ value, the iMNP and Gadovist^®^ were characterized as 7.5 mM[Fe]^-1^ and 3.5 s^-1^·mM[Gd]^-1^, respectively. iMNP was similarly higher enhancement capability for *T*_2_ MRI contrast agent. CT contrast phantom image of iMNP possessing Bi ions was carried out for sensitivity measurement, and the CT numbers (Hounsfield unit, HU) compared with clinically used Telebrix^®^ 30 (TB) CT contrast imaging agent (B). The iMNP revealed high HU values at the same concentration of Bi and I, which were also calculated by ICP-AES after detection, as much as 4.5-fold HU value per same concentration (mM) of ion. We believe that the particulate form (iMNP) showed enhanced intensity owing to the restricted rotational mobility as a mobile solid property.


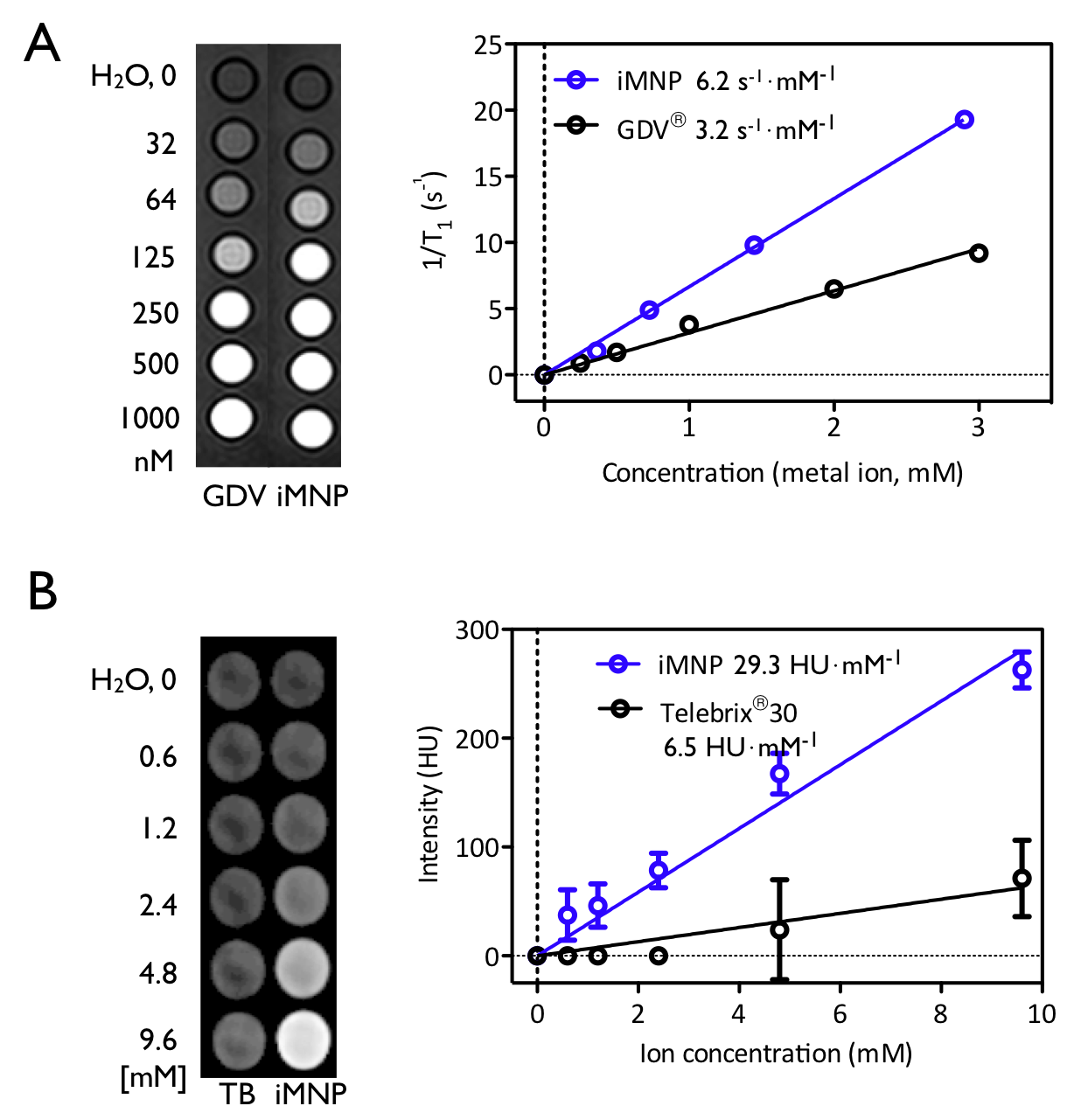


**Figure S5.** Measurement of ion leakage of the iMNPs at various pH or human serum conditions (A). iMNP were incubated in various solutions and then isolated by centrifugation (13,000 rpm, 15 min). The supernatant was carried out determining ions concentration by ICP-AES. All samples showed < 3 % release from the particle. After iMNP-EGFR particle treatment, the HepG2 cell viability was determined using a typical cell proliferation assay (MTT) and showed a survival rate of > 90 % at various concentrations. The biocompatibility of iMNP was investigated by assessing cellular organelle functions. Expression levels of VDAC1 (voltage-dependent anion channel-1), ATG5 (autophagy protein 5), SOD2 (superoxide dismutase 2 mitochondrial), and Cyto.c (cytochrome complex) protein biomarkers of inner cell signaling and mitochondrial function were determined by Western blot analysis (B). All treatment (i: only cell, ii: iMNP, and iii: iMNP-EGFR) samples had similar protein expression levels and did not show any significant intensity changes.


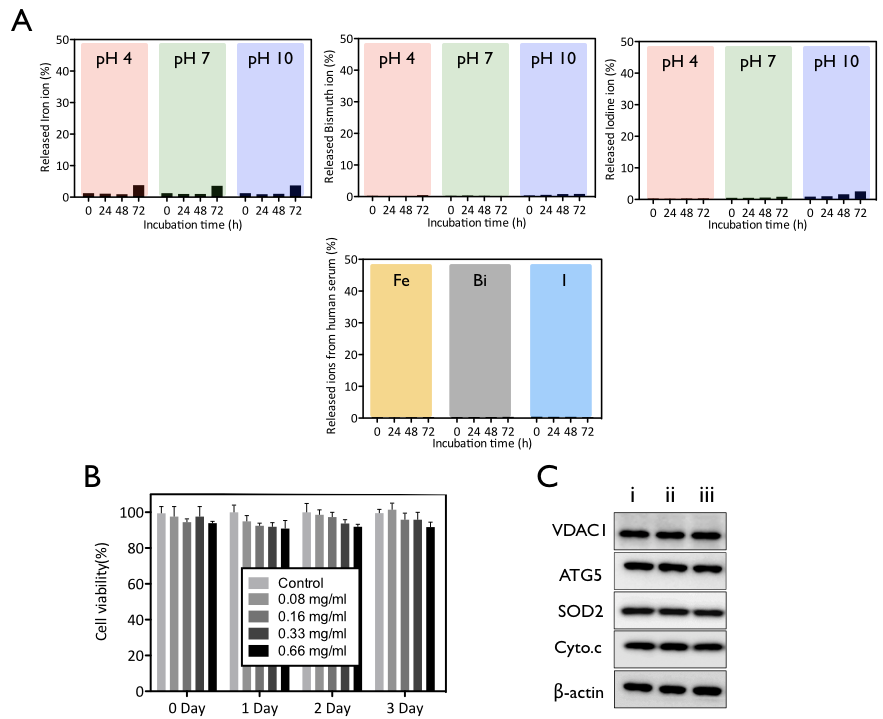


**Figure S6.** Characterization of EGFR expression levels by Western blot analysis. HepG2 cells were designed to over express EGFR (positive control cell line), while MCF7 and NIH-3T3 showed similar expression levels as a negative control cell line. For the EGFR antibody control HepG2 cells, bare iMNP did not show red fluorescence intensity and the iMNP-EGFR treated cells showed the most red fluorescence (B and C). These results suggest that the prepared iMNP-EGFR solution can be effectively used for the specific targeting of EGFR-positive HepG2 cancer cells.


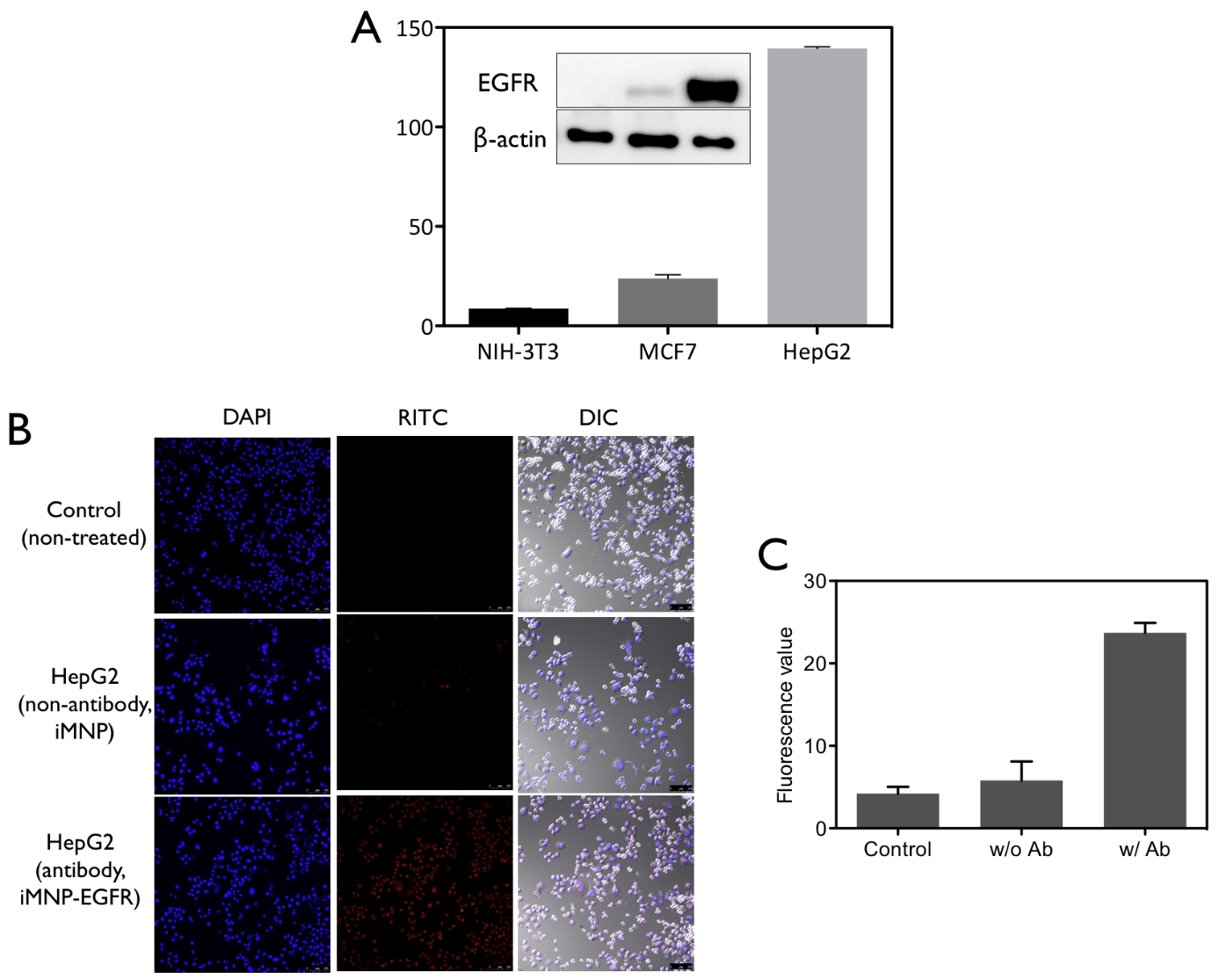


**Figure S7.** Various imaging data for iMNP without antibody modification as a control. After intravenous administration of iMNP particles cancer mouse, various imaging results were obtained (A: *T*_1_-*w* MRI axial view, B: micro-CT coronal view, and C: micro-SPECT coronal projection view). The white circle indicats the implanted HCC tumor in the liver.

**
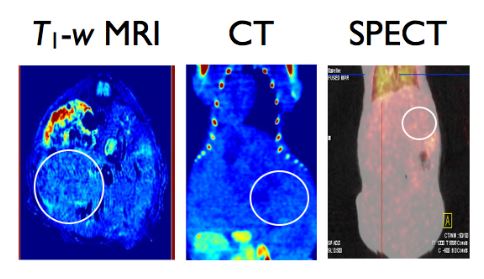
**

**Figure S8.** As a fundamental toxicological study, MNP and iMNP-EGFR preparations were injected into normal mice and then body weight was measured for 15 days (A). No significant difference in body weight compared PBS injected control mouse was observed. After 24 hr of treatment (B), the MNP injected mice revealed abnormal organ color, especially in the lung (Lu), liver (L), and spleen (S). We determined the organ weight for the various treated mice but found the weight similar to the control (C).


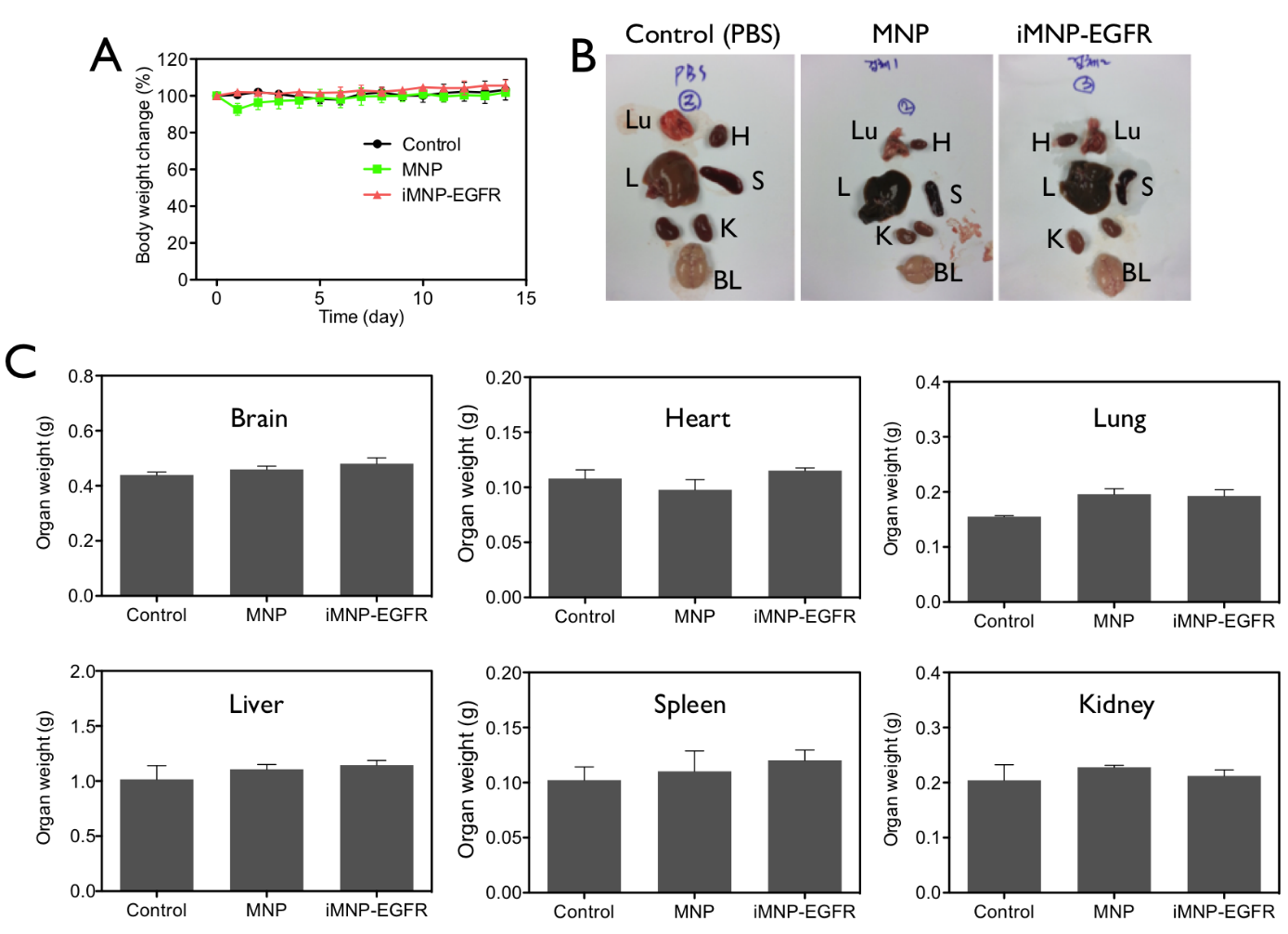


**Figure S9.** Microscopic image of mouse model organs after iMNP-EGFR nanoparticle administration (left image), and the liver, spleen, and lung showed abnormal black color due to the iMNP-EGFR nanoparticles. From the histopathological analysis, the organs showed normal morphology after staining with hematoxylin and eosin (H&E, right). The implanted HepG2 liver cancer tissue was subjected to immunohistochemical staining for EGFR-positive cells (dark brown indicates the cancer region, right bottom image).


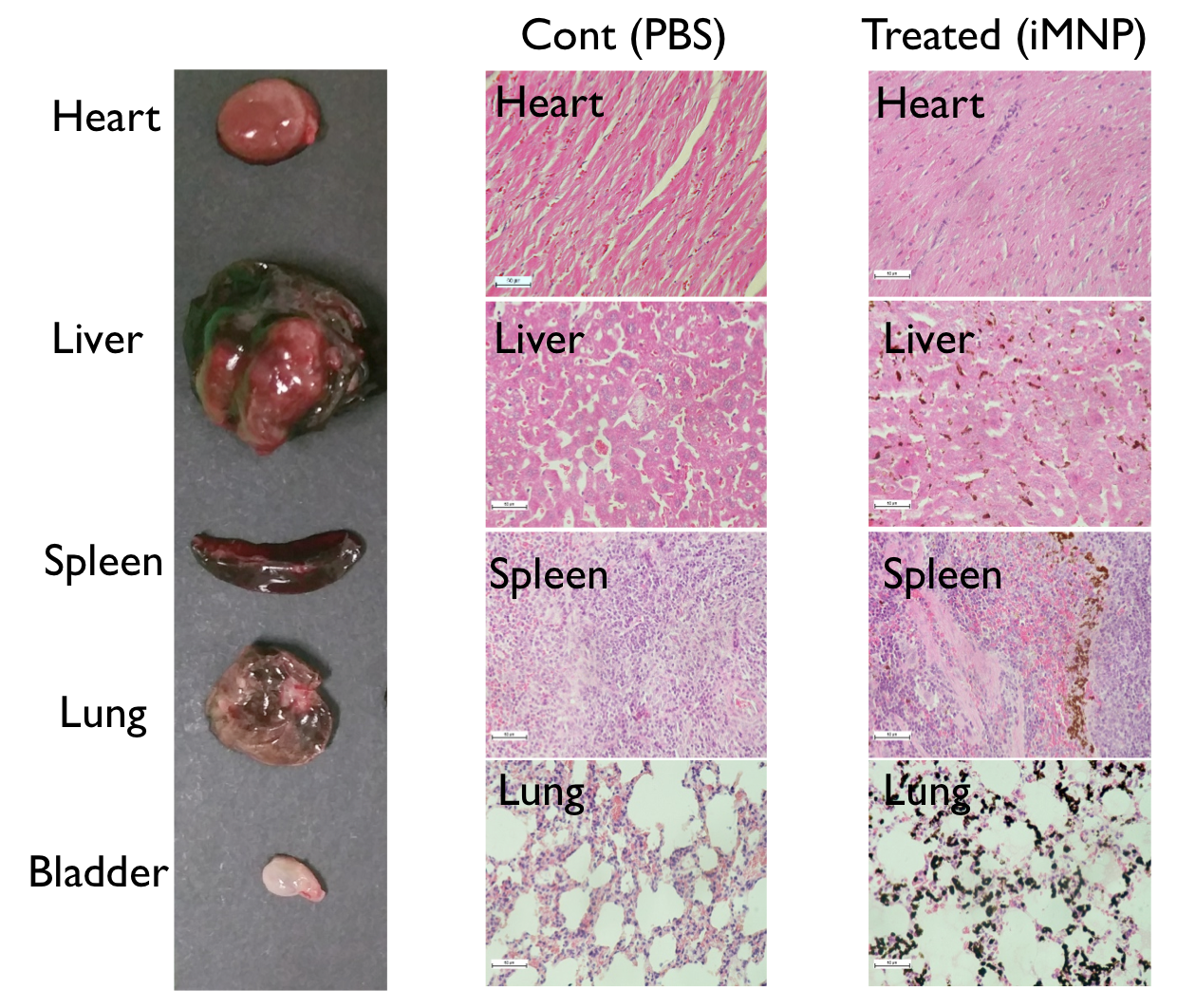

Supplement: Supplementary file 1 — Additional file 1. Iodination of MNP using an IODO-GEN tubes. STEM analysis data for ionized MNPs. Characterization data for iMNPs. Phantom study data for iMNPs in solution. Cellular toxicity assessment after iMNP treatment. Characterization data for the expression levels of EGFR. In vivo toxicological data after administration of iMNPs. [file 12951_2017_304_MOESM1_ESM.docx]
